# Ferroptosis-related factors in the substantia nigra are associated with Parkinson’s disease

**DOI:** 10.1038/s41598-023-42574-4

**Published:** 2023-09-16

**Authors:** Lei Liu, Yange Cui, Yan-Zhong Chang, Peng Yu

**Affiliations:** Ministry of Education Key Laboratory of Molecular and Cellular Biology, Hebei Key Laboratory of Animal Physiology, Biochemistry and Molecular Biology, College of Life Sciences, Hebei Normal University, No. 20 Nan’erhuan Eastern Road, Shijiazhuang, 050024 Hebei Province China

**Keywords:** Cell death in the nervous system, Diseases of the nervous system

## Abstract

Ferroptosis is an iron-dependent, lipid peroxidation-driven cell death pathway, while Parkinson’s disease (PD) patients exhibit iron deposition and lipid peroxidation in the brain. Thus, the features of ferroptosis highly overlap with the pathophysiological features of PD. Despite this superficial connection, the possible role(s) of ferroptosis-related (Fr) proteins in dopaminergic neurons and/or glial cells in the substantia nigra (SN) in PD have not been examined in depth. To explore the correlations between the different SN cell types and ferroptosis at the single-cell level in PD patients, and to explore genes that may affect the sensitivity of dopaminergic neurons to ferroptosis, we performed in silico analysis of a single cell RNA sequence (RNA-seq) set (GSE178265) from the Gene Expression Omnibus (GEO) database. We identified differentially expressed genes (DEGs) in the different cell types in the human SN, and proceeded to perform enrichment analysis, constructing a protein–protein interaction network from the DEGs of dopaminergic neurons with the Metascape database. We examined the intersection of Fr genes present in the FerrDb database with DEGs from the GSE178265 set to identify Fr-DEGs in the different brain cells. Further, we identified Fr-DEGs encoding secreted proteins to implicate cell–cell interactions in the potential stimulation of ferroptosis in PD. The Fr-DEGs we identified were verified using the bulk RNA-seq sets (GSE49036 and GSE20164). The number of dopaminergic neurons decreased in the SN of PD patients. Interestingly, non-dopaminergic neurons possessed the fewest DEGs. Enrichment analysis of dopaminergic neurons’ DEGs revealed changes in transmission across chemical synapses and ATP metabolic process in PD. The secreted Fr-DEGs identified were ceruloplasmin (CP), high mobility group box 1 (HMGB1) and transferrin (TF). The bulk RNA-seq set from the GEO database demonstrates that CP expression is increased in the PD brain. In conclusion, our results identify CP as a potential therapeutic target to protect dopaminergic neurons by reducing neurons’ sensitivity to ferroptosis.

## Introduction

Parkinson’s disease (PD) is the second most prevalent neurodegenerative disease (ND) in the elderly after Alzheimer's disease (AD)^1,2^. The clinical characteristics of PD comprise motor disorders, such as static tremor, muscle stiffness, motor retardation, gait disorders and non-motor symptoms, such as reduced sense of smell, mental disorders, sleep disorders, cognitive disorders^3^. As a result, the daily activities and self-care behavior of PD patients are severely impaired, markedly affecting the quality of life and increasing the burden on families and society. Despite decades of effort, the pathogenesis of Parkinson’s disease remains enigmatic; strategies for effective treatment targets are still urgently needed.

The main characteristic of PD is the reduction or disappearance of dopaminergic neurons in the substantia nigra (SN), with the depletion of dopamine in the striatum and the emergence of Lewy bodies with α-synuclein aggregation as main components. Interestingly, these features are also accompanied by systemic iron deposition and lipid peroxidation in SN^4,5^. Ferroptosis is a distinct form of cell death, driven by iron-dependent lipid peroxidation, that is morphologically and mechanistically distinct from apoptosis and other known cell death pathways^6^. Previous studies have shown a high degree of overlap between ferroptosis and PD pathophysiological features, such as iron overload, increased lipid peroxidation, reduced glutathione levels, downregulation of the cystine-glutamate antiporter system (System xc-), and decreases in DJ-1 and coenzyme Q10 (CoQ10)^7^. It is newly reported that iron is correlated with parkinsonism symptoms in a PD mouse model with mutation in *Pla2g6* gene (phospholipase A2 group VI), and that iron-catalyzed lipid oxidation modification reduces glutathione peroxidase 4 activity and content^8^, which is related closely to PD. These findings suggest that ferroptosis may be important in the progression of PD.

The main and effective treatment for PD is to increase dopamine nerve conduction^9^. The combined use of L-DOPA agonists and decarboxylase inhibitors remains the “gold standard” in the treatment of PD^10^. Nevertheless, as the disease progresses, due to a lack of neuroprotective drugs, there remains a great unmet need for effective neuroprotective or disease-modifying therapies^11^. Although there is high correlation between ferroptosis and PD, there have been no conclusive reports describing the involvement of ferroptosis-related (Fr) genes in dopaminergic neurons and/or glial cells. Thus, we set out to explore the possible involvement of ferroptosis in PD, which may open the door to future development of ferroptosis-based disease treatment strategies to delay PD progression.

In this study, we used single-cell data from the GEO database to conduct a bioinformatic analysis of the SN of PD patients. Differentially expressed genes (DEGs) of different cell types in the SN of were identified in the patients. We then performed DEG enrichment analysis of dopaminergic neurons and constructed a protein–protein interaction (PPI) network. Meanwhile, we screened the FerrDb v2 database for Fr DEGs. We identified the secreted proteins in the Fr-DEGs to explore the interactions between cells. The secretory proteins were verified using the bulk RNA-seq data (GSE49036, GSE20164) of the GEO database. Together, our data reveal correlations between different cell types and ferroptosis at the single-cell level, and identify factors that may affect the sensitivity of dopaminergic neurons to ferroptosis.

## Materials and methods

### Data collection

Single cell data and annotated information was downloaded and viewed from GEO and the Broad Institute’s Single Cell Portal. We used the GEO accession number GSE178265 to download single-cell data in the GEO database (https://www.ncbi.nlm.nih.gov/geo/). Annotated information of cell types was also downloaded from the Broad Institute’s Single Cell Portal (https://singlecell.broadinstitute.org/single_cell/study/SCP1768/). PD-related bulk RNA-seq data (GSE49036, GSE20164) was downloaded and viewed in the GEO database. Since the data used were all from public databases, patient consent or ethics committee approval were not required.

### Cell type annotation and DEGs analysis

The data were read and processed using the R software package, Seurat v4.2.0^12^. The data on human SN cells was extracted by adding the downloaded metadata from all cells to meta.data in SeuratObject^13^. The quality control criteria for the data were as follows: cells with fewer than 10% reads mapping to mitochondrial genes and greater than 650 unique molecular identifiers (UMI) were retained. For the objects in SeuratObject, the NormalizeData function of the Seurat package was used for normalization, FindVariableFeature was used for searching highly variable genes and ScaleData function was centralized (with the default parameter). For the annotation of cell types, we followed the results uploaded by Prof. Macosko’s group on the single cell portal^14^ to extract seven cell types: dopaminergic neurons, non-dopaminergic neurons, astrocytes, oligodendrocytes, oligodendrocyte progenitor cells (OPCs), endothelial cells/pericytes and microglia/macrophages. We used the FindMarkers function of the Seurat package to group by disease status; DEG analysis was performed using the ‘wilcox’ algorithm with a logfc.threshold value of 0.4.

### Screening of Fr-DEGs in DEGs of SN cells from PD patients

The Fr-gene symbol collected in this study were downloaded from the FerrDb v2 database (http://www.zhounan.org/ferrdb/current/)^15^. The DEGs were first screened based on a p_val_adj less than 0.05 (adjusted p-value, based on Bonferroni correction using all genes in the dataset) and an avg_log_2_FC above 0.4 (avg_log_2_FC: log fold-change of the average expression between PD and control groups; positive values indicate that the gene is more highly expressed in the PD group). The online software, VENNY v2.1 (https://bioinfogp.cnb.csic.es/tools/venny/)^16^ was used to intersect the DEGs with all ferroptosis-related genes, redrawing the Venn Diagram with improved clarity and accuracy.

### Functional enrichment and PPI analysis

The Metascape database (https://metascape.org) is a web-based portal that integrates more than 40 biological information databases—through a simple interface for “one-click”, quick analysis, comprehensive data analysis can be easily obtained^17^. We used Metascape to conduct a multiple database (including GO Biological Processes, KEGG Pathway, a Reactome gene set and WikiPathways) enrichment analysis, and construct a PPI network.

### Screening of secreted proteins in Fr-DEGs

The intersection of DEGs and Fr-genes was used to screen for Fr-DEGs. We used the gene function annotations from the UniProt and Protein Altas databases in Metascape, and screened for genes that encode secreted proteins.

### Screening for DEGs in microarray data

The R software package, limma (v3.52.4)^18^, was used to conduct differential gene analysis on two PD microarray datasets (GSE49036, GSE20164). The genes with p-value ≤ 0.05 and logFC ≥ 0.5 were considered differentially expressed, since we found the number of differential genes in the transcriptome sequencing of PD patients is relatively less, and we think small changes in protein expression in the brain can lead to dysregulation of the neural environment.

## Results

### Cell types and their proportion in the SN of PD patients

**Figure 1:**
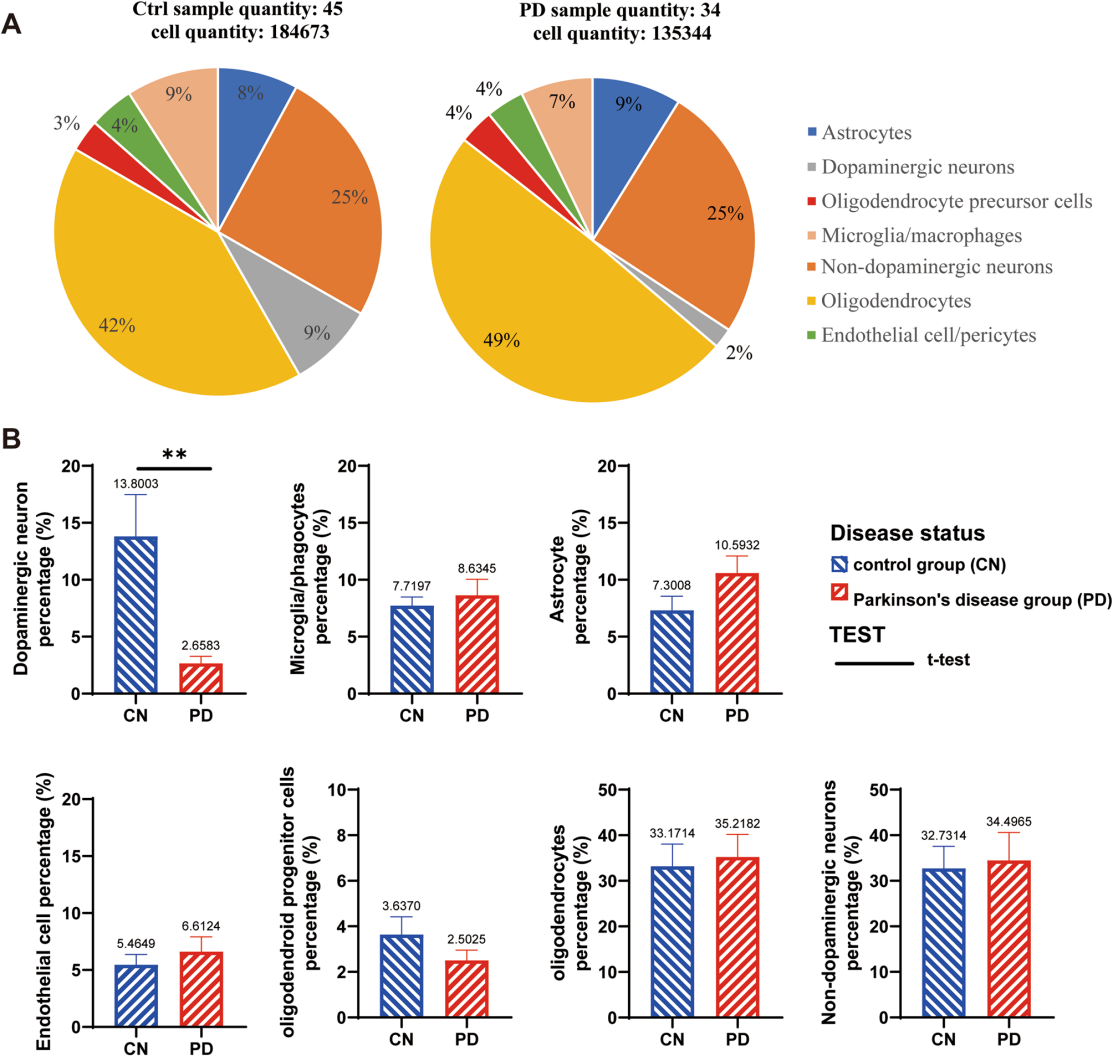
Cell types and proportions in the SN of PD patients and the control group (CN). A shows the combining multiple samples together for statistical analysis. A total of 184,673 cells from 45 CN people were screened in the control group, and the whole number of each cell types was calculated to the total cell population (184,673 cells). While 135,344 cells from 34 PD patients were screened in the PD group, in which the proportion of each cell types was calculated to the whole population (135,344 cells). B is the statistical analysis of the proportion of cells in each individual sample with T-TEST, there are 45 CN samples and 34 PD patients used for statistical analysis, but the samples with data of 0% were excluded in charts. **p < 0.01.

Based on the cell annotation results on the Single Cell Portal uploaded by Prof. Macosko’s group, cells were extracted and classified into different types from human SN samples (Fig. 1A). After 135,344 cells were extracted from 34 PD samples and 184,673 cells were extracted from 45 control samples. We obtained a total of seven major groups of cells, which were dopaminergic neurons, non-dopaminergic neurons, astrocytes, oligodendrocytes, OPCs, endothelial cells/pericytes and microglia/macrophages. By statistical analysis of the proportion of cells in each sample, we found that only dopaminergic neurons were significantly decreased in the SN pars compacta in PD patients, and that other six groups of cells were not changed obviously (Fig. 1B).

### Identification of DEGs between the PD and control groups

**Figure 2:**
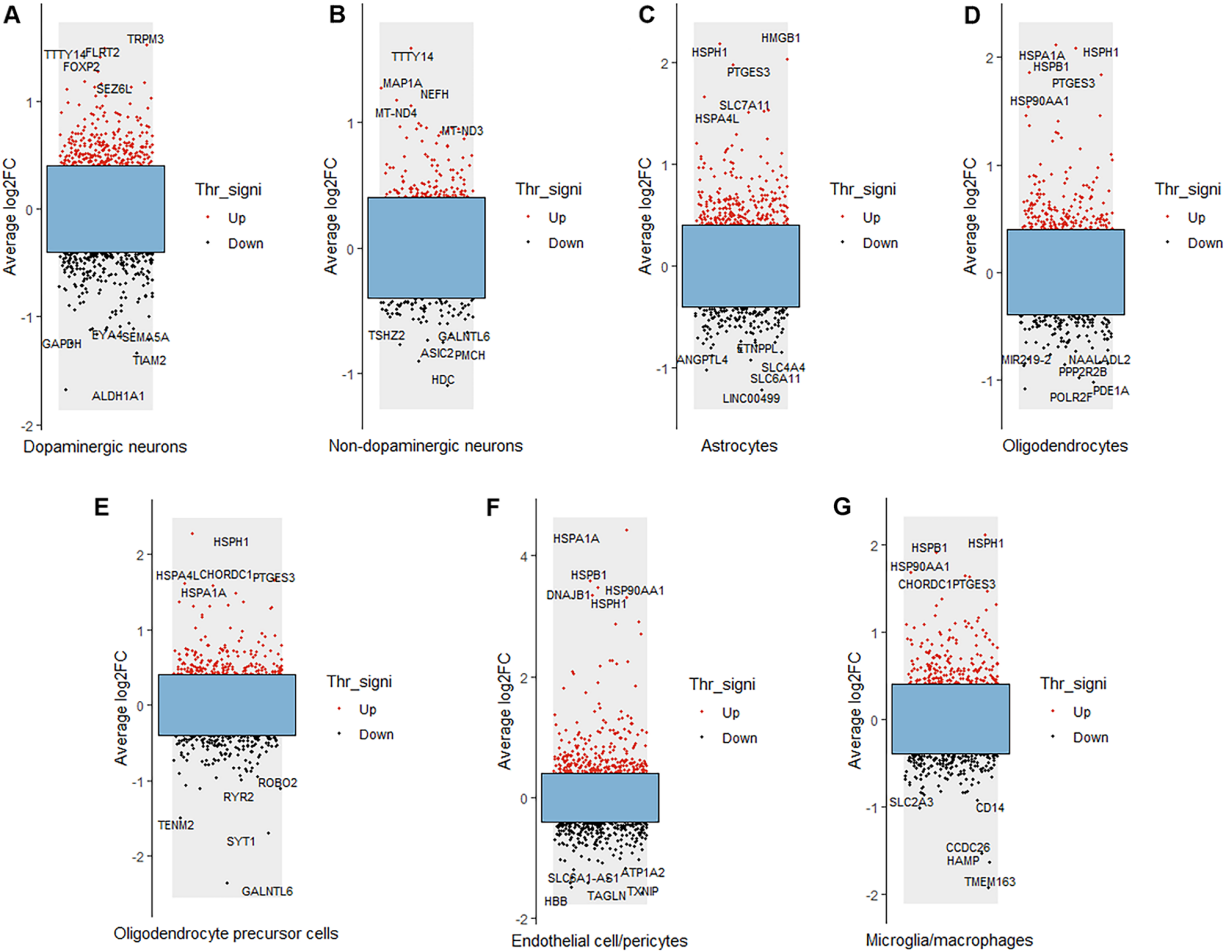
Results of DEG analysis of each cell type in PD. Red dots represent up-regulated genes; black dots represent down-regulated genes. Average log2FC indicates average log2-fold change, and Thr_signi represents the threshold value and significance. The five most up-regulated genes and the five most down-regulated genes in each cell type are provided.

The results of differential gene analysis of each cell type in the SN of PD patients and controls are shown in Fig. 2. The five DEGs with the greatest up- and down-regulation changes are marked in each type of cell. The five most upregulated genes in dopaminergic neurons were TRPM3, TTTY14, FLRT2, FOXP2 and SEZ6L, while the five most downregulated genes were GAPDH, EYA4, SEMA5A, TIAM2 and ALDH1A1 (Fig. 2A). The five most up-regulated genes in non- dopaminergic neurons were TTTY14, MAP1A, NEFH, MT-ND4 and MT-ND3, while the five most down-regulated genes were TSHZ2, GALNTL6, ASIC2, PMCH and HDC (Fig. 2B). The five most upregulated genes in astrocytes were HMGB1, HSPH1, PTGES3, SLC7A11 and HSPA4L, while the five most downregulated genes were ANGPTL4, ETNPPL, SLC4A4, SLC6A11 and LINC00499 (Fig. 2C). The five most upregulated genes in oligodendrocytes were HSPH1, HSPA1A, HSPB1, PTGES3 and HSP90AA1, while the five most downregulated genes were NAALADL2, MIR219-2, PPP2R2B, PDE1A and POLR2F (Fig. 2D). The five most upregulated genes in OPCs were HSPH1, CHORDC1, HSPA4L, PTGES3 and HSPA1A, while the five most downregulated genes were ROBO2, RYR2, TENM2, SYT1 and GALNTL6 (Fig. 2E). The five most upregulated genes in endothelial cell/pericytes were HSPA1A, HSPB1, DNAJB1, HSPH1 and HSP90AA1, while the five most downregulated genes were ATP1A2, TXNIP, SLC6A1-AS1, TAGLN and HBB (Fig. 2F). The five most upregulated genes in microglia/macrophages were HSPH1, HSPB1, HSP90AA1, CHORDC1 and PTGES3, while the five most downregulated genes were CD14, SLC2A3, CCDC26, HAMP and TMEM163 (Fig. 2G).

### Screening of ferroptosis-related DEGs in the seven cell types

We downloaded ferroptosis related gene from FerrDb v2. Among the Fr-genes, genes that have been verified in humans were selected, and duplicate genes were removed, based on gene symbol. Among these, 5 genes (AQP3, AQP5, AQP8, DAZAP1, NT5DC2) were not annotated in the verified tissues, but were confirmed to be related to ferroptosis in humans by consulting the literature^19^. Finally, 401 Fr-genes were obtained through screening.

**Figure 3:**
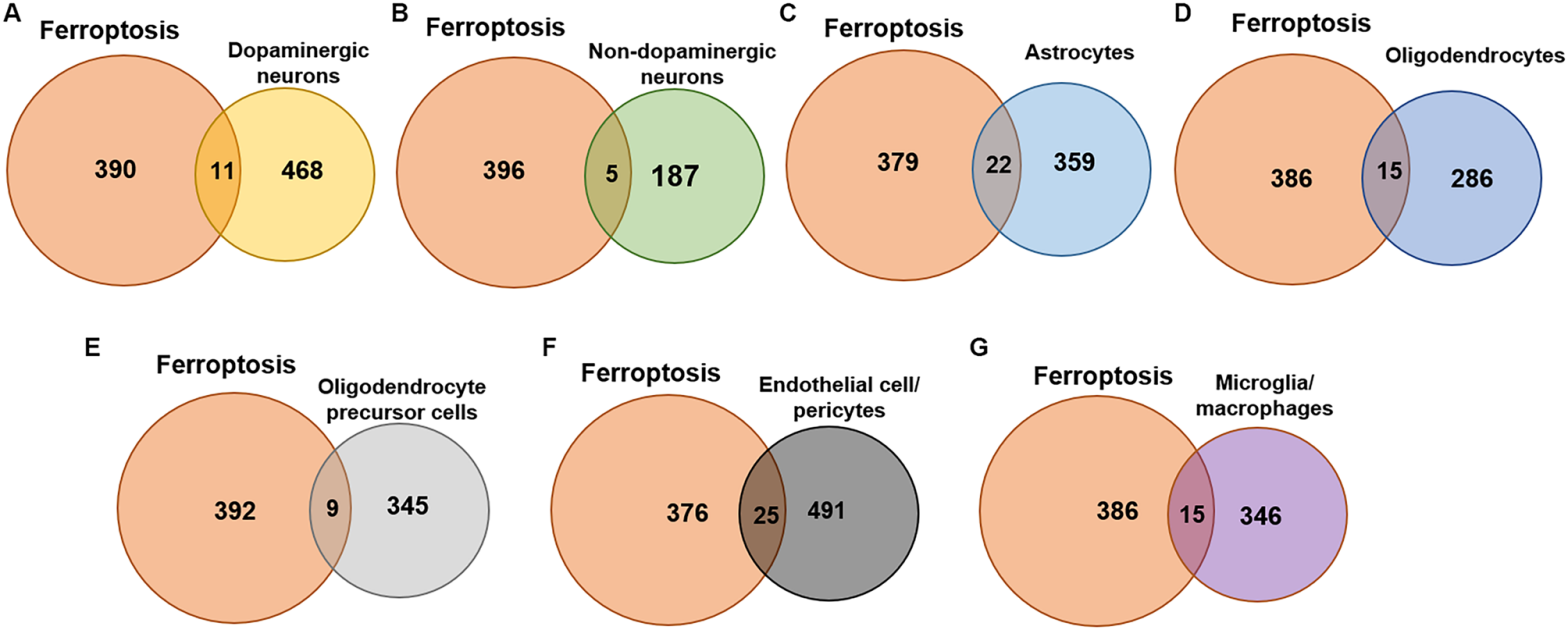
Fr-DEGs in various cell types.

The Fr-DEGs of each type of cell were identified using the intersection method. There were 479 DEGs in dopaminergic neurons, with 11 Fr-DEGs identified (Fig. 3A). There were 192 DEGs in non-dopaminergic neurons, with 5 Fr-DEGs identified (Fig. 3B). The difference in numbers of DEGs between dopaminergic neurons and non-dopaminergic neurons is indicative of the specific damage to dopaminergic neurons in PD. Astrocytes showed 381 DEGs, with 22 Fr-DEGs identified (Fig. 3C). There were 301 DEGs in oligodendrocytes, with 15 Fr-DEGs identified (Fig. 3D). There were 354 DEGs in OPCs, with 9 Fr-DEGs identified (Fig. 3E). There were 516 DEGs in endothelial cell/pericytes, with 25 Fr-DEGs identified (Fig. 3F). There were 361 DEGs in microglia/macrophages, with 15 Fr-DEGs identified (Fig. 3G). All the genes with altered expression are shown in Table 1. Although FTH expression decreased in dopaminergic neurons, the change was not shown due to a p_val_adj > 0.05.

**Table 1:** Expression of Fr-DEGs in each type of cell.

DA neurons		Non-DA neurons		Astrocytes		Oligodendrocytes		Oligodendrocyte precursor cells		Endothelial cell/pericytes		Microglia/macrophages	
Gene symbol	Average log2FC	Gene symbol	Average log2FC	Gene symbol	Average log2FC	Gene symbol	Average log2FC	Gene symbol	Average log2FC	Gene symbol	Average log2FC	Gene symbol	Average log2FC
GPX4	− 0.60	HSPB1	0.98	SLC7A11	1.67	FTH1	− 0.77	HSPB1	1.48	SLC7A11	0.55	HSPB1	1.90
HSPB1	0.49	OIP5-AS1	0.46	HSPB1	1.06	SLC7A11	0.42	STAT3	0.45	HSPB1	3.58	SQSTM1	0.78
AR	0.42	HMGB1	0.61	SQSTM1	0.61	HSPB1	1.86	BEX1	− 0.62	SQSTM1	0.95	PLIN2	0.46
BEX1	− 1.11	SNCA	0.43	MT1G	0.68	SQSTM1	0.77	PEBP1	− 0.53	HSPA5	0.73	NEDD4L	0.51
MGST1	− 0.57	MIB1	0.43	HSPA5	0.59	TF	− 0.50	HMGB1	1.36	ATF4	0.71	NUPR1	0.94
RPL8	− 0.41			STAT3	0.46	NUPR1	0.87	DNAJB6	0.83	CDKN1A	0.48	FTL	0.84
PEBP1	− 0.95			CD44	0.64	SOX2	0.67	CIRBP	− 0.50	JUN	0.43	CYBB	− 0.43
HMGB1	0.85			SESN2	0.61	HMGB1	0.97	LIFR	0.48	PLIN2	1.09	TGFBR1	− 0.42
SLC38A1	0.58			ARNTL	0.46	DNAJB6	0.89	MIB1	0.80	PRDX1	0.45	HMGB1	0.72
KDM5A	0.44			CP	0.57	SLC38A1	0.55			NUPR1	0.97	DNAJB6	0.93
MIB1	0.58			IDH2	− 0.63	SNCA	0.50			BEX1	− 0.40	ELOVL5	0.43
				NUPR1	0.87	KDM5A	0.43			FTL	1.32	SNCA	0.73
				NEAT1	1.01	CIRBP	− 0.73			ACSL4	0.44	PRKCA	− 0.55
				PDK4	− 0.52	TRIM26	0.46			SAT1	0.52	CIRBP	− 0.48
				FTL	0.58	MIB1	0.84			HMGB1	0.52	MIB1	0.94
				HMGB1	2.19					ATF3	0.75		
				DNAJB6	0.89					DNAJB6	1.36		
				ELOVL5	0.42					ELOVL5	0.43		
				SLC38A1	0.68					PTEN	− 0.65		
				DDR2	0.84					MTCH1	0.71		
				CIRBP	− 0.54					KLF2	− 0.71		
				MIB1	1.08					CIRBP	− 0.70		
										TRIM26	0.59		
										NDRG1	0.71		
										MIB1	1.36		

Fr-DEGs: ferroptosis-related differentially expressed genes, DA: dopaminergic; log2FC: log fold-change. Average log2FC > 0 indicates that the gene is up-regulated, while Average log2FC < 0 indicates that the gene is down-regulated. Adjusted *p*-value < 0.05 was identified as a DEG.

### Enrichment analysis of DEGs in dopaminergic neurons and construction of a PPI network

**Figure 4:**
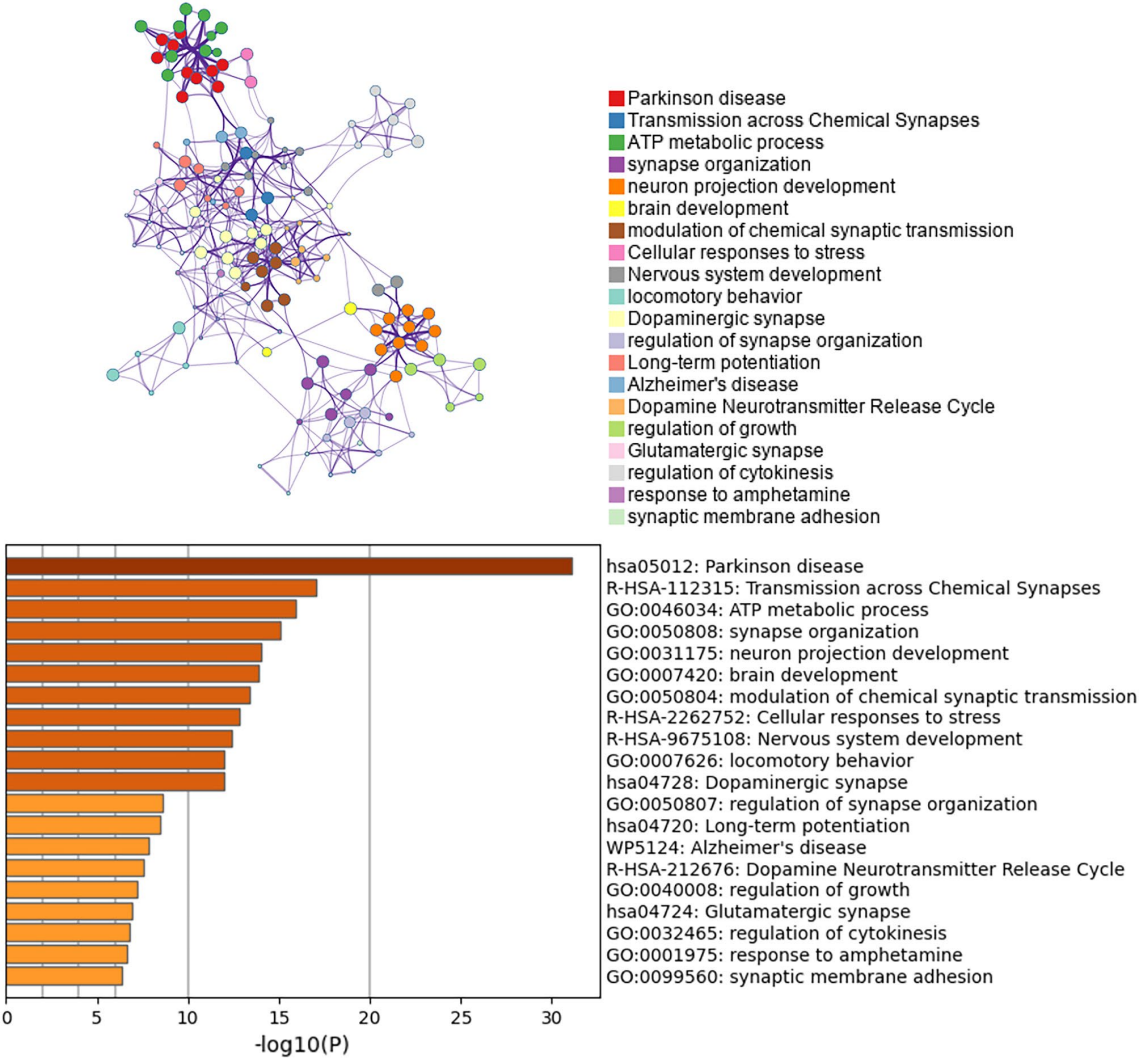
Hierarchical clustering results and clustering trees for functional enrichment of dopaminergic neuron DEGs. Only the *p*-value information is shown in the histogram, which can be viewed against the dendrogram.

**Figure 5:**
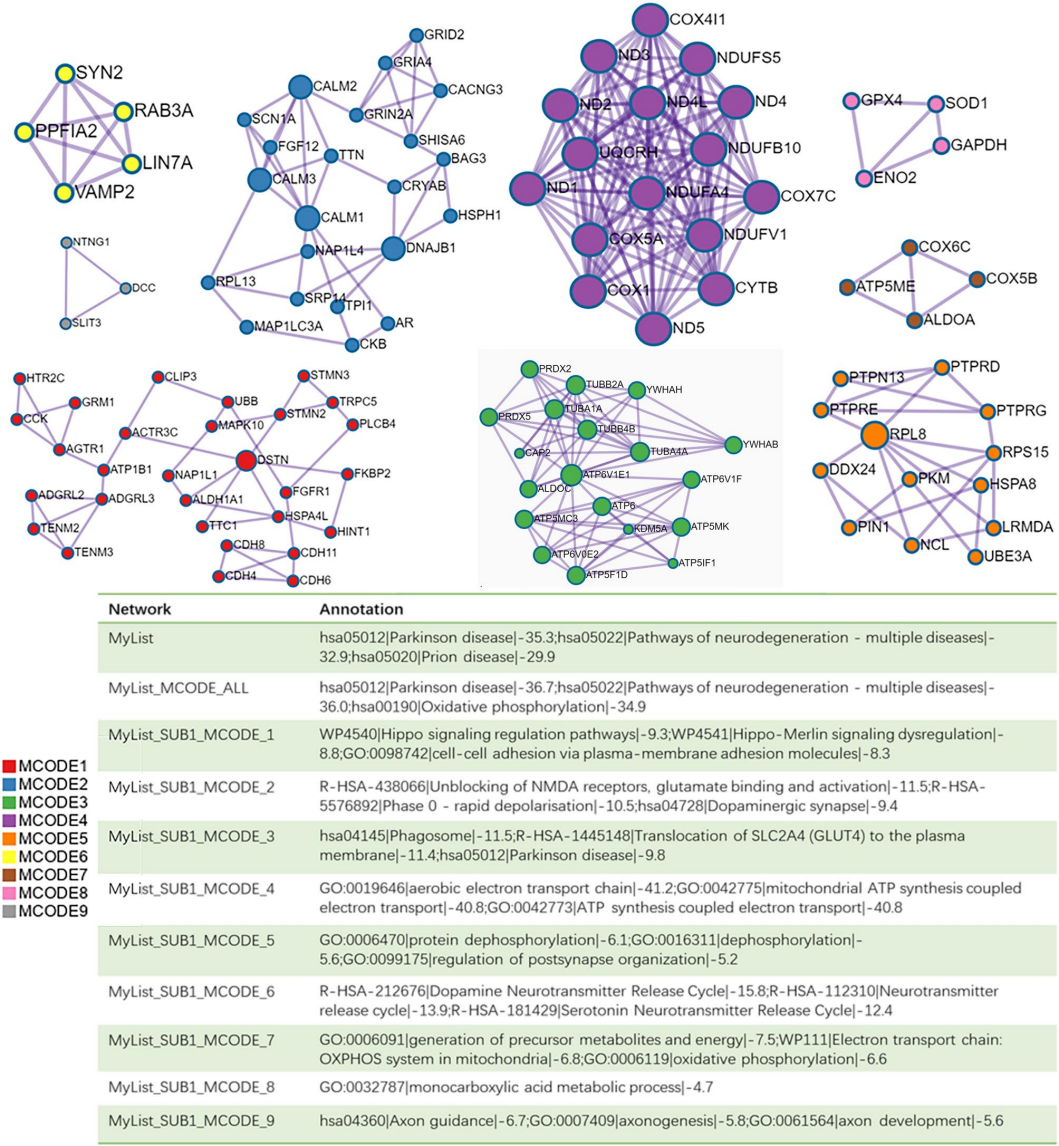
PPI network and MCODE component analysis results. Genes that do not interact with other genes in the list of DEGS are not provided. The PPI network shows genes that have cooperative relationships with other genes, and also possess MCODE components. MCODE reveals closely connected molecular complex regions in the PPI network.

The loss of dopaminergic neurons in the SN is a hallmark feature of the pathophysiology of PD. Therefore, we conducted enrichment analysis and PPI network construction of the DEGs of dopaminergic neurons. The enrichment analysis of the dopaminergic neuron DEGs mainly revealed changes in ATP metabolism process, cell response to stress, motor behavior, dopaminergic synapses, dopamine neurotransmitter release cycle and long-term potential difference in the dopaminergic neurons of PD patients (Fig. 4). The Cytoscape classic plug-in MCODE (molecular complex detection) of Metascape can be used to discover closely connected molecular complex regions in a PPI network. We performed functional enrichment analysis on the identified sub-networks to further clarify the role of these potential complexes in life regulation. The PPI network shows genes that have cooperative relationships with other genes, in the context of MCODE components (Fig. 5). Overall, the MCODE data not only show the relationships of the differential gene expression in Parkinson’s and various NDs, but also specifically demonstrate enrichment of the oxidative phosphorylation pathway.

### Identification of secreted proteins from Fr-DEGs in all cells

Interactions between cells is a key feature in the cellular activities of multicellular life^20^. We hypothesized that a secreted protein between cells contributes to the loss of neurons in PD by affecting the sensitivity of dopaminergic neurons to ferroptosis. To test this, we annotated the gene functions of Fr-DEGs in all cell types, and then screened for genes encoding secreted proteins. Three DEGs encoding secreted proteins (HMGB1 [high-mobility group box 1 protein], CP [ceruloplasmin], TF [transferrin]) were found in PD patients. Among these genes, the expression of CP increased in astrocytes, the expression of HMGB1 increased in all types of cells, and the expression of TF decreased in oligodendrocytes (Table 2).

**Table 2:** Identification of secreted proteins from the Fr-DEGs in all cell types.

Protein name	Gene symbol	Secreted (UniProt)	Secreted (Protein Altas)	Variation tendency	Ferroptosis (FerrDb V2)
High mobility group box 1	HMGB1	Yes	Yes	Up	Driver
Ceruloplasmin	CP	Yes	Yes	Up	Suppressor
Transferrin	TF	Yes	Yes	Down	Driver/Suppressor

The resulting genes expressing secreted proteins were identified using UniProt and the Protein Altas database. The expression of CP increased; the expression of HMGB1 increased; and the expression of TF decreased.

### Screening for Fr-DEGs encoding secreted proteins in microarray data

Using the bulk RNA-seq data in the GEO database, we verified the altered expression of the three genes, HMGB1, CP, and TF, in the SN of PD patients. Gene changes at the SN level are congruent with the hypothesis that dopaminergic neurons are affected by these proteins. The increase in CP expression was verified in the GSE49036 and GSE20164 datasets (Table 3). However, there were no significant verified changes in HGB1 or TF, which may be due to technical differences between the screening methods. This implicated that changes at the single-cell level don't necessarily reflect changes at the overall tissue level.

**Table 3:** Changes in HMGB1, CP and TF at the SN level in PD.

GEO datasets	PD samples	CON samples	Platforms	HMGB1 logFC	CP logFC	TF logFC
GSE49036	15	8	GPL570	Unknown	0.54	Unknown
GSE20164	6	5	GPL96	Unknown	1.60	Unknown

The identified Fr-DEGs were evaluated at the holistic level in the SN of PD patients using the GEO data set. LogFC > 0 indicates that the gene is up-regulated, whereas logFC < 0 indicates that the gene is down-regulated.

## Discussion

PD has become a major burden on families and society, especially with the increases in the aging population worldwide. There is still an urgent need for treatment strategies and modulating therapies to control PD^21^. Ferroptosis has been found to be involved in many diseases, recently garnering increasing attention. In the past couple decades, investigations into PD pathology have revealed some distinct features, such as iron accumulation, lipid peroxidation, glutathione depletion, CoQ10 deficiency, glutathione peroxidase (Gpx4) reduction, mitochondrial lesions, among others^7^. Interestingly, these characteristics also strongly correlate with ferroptosis, raising the possibility that ferroptosis is involved in the loss of dopaminergic neurons in PD. With the advancements in omics technology and the development of bioinformatics, it now becomes possible to closely examine the etiological mechanisms and conceive prevention strategies of PD in silico. In the present study, we performed bioinformatics analysis using a dataset from single-cell transcriptome sequencing of the SN in PD patients. By annotating cell types, we found a significantly decrease in dopaminergic neurons. This is the most significant pathological feature of PD, suggesting that seeking dopaminergic neuronal protective strategies may be the most important means to treat PD.

In exploring the characteristics of the DEGs in different types of cells in the PD brain, we found that HAMP appeared among the five genes with the greatest reduced expression in oligodendrocytes. The HAMP gene encodes pro-hepcidin, a small protein consisting of 84 amino acids that is cleaved to generate mature hepcidin (25 amino acids)^22^. Hepcidin binds to the only known cellular iron export protein, ferroportin1 (FPN1), leading to its internalization and degradation^23^. This decreased expression of hepcidin in oligodendrocytes warrants validation and examination of the role of this iron metabolism regulator in the pathogenesis of PD. Meanwhile, the expression of ferritin heavy chain (FTH) was decreased in oligodendrocytes. As oligodendrocytes are the main sites of iron storage in the brain^24^, the stimulation of FPN1-mediated iron export via decreased hepcidin is expected to increase free extracellular iron, which may, in turn, result in oxidative stress in the SN in PD patients.

The genes of different cell types exhibited marked differences in magnitude. Non-dopaminergic neurons exhibited the lowest number of differential genes, while dopaminergic neurons exhibited extensive alterations in gene expression. Dopaminergic neurons in the SN of PD face greater stressors compared to non-dopaminergic neurons. This may explain why the loss of dopaminergic neurons in the SN is the main pathological feature in PD, which is significantly different from AD, in which the loss of neurons in the medial temporal lobe is dominant^25^. Intriguingly, there are numerous iron metabolism-related genes altered in the SN of PD patients, suggesting that dysregulated iron metabolism may participate in the pathogenesis of PD. It has also been reported that iron, rather that zinc or copper, accumulates in the SN with aging^26^. In addition, the functional enrichment analysis of the DRGs in dopaminergic neurons that revealed pathways related to PD, also indicated there are additional important pathways, such as ATP metabolic process, cellular response to stress, and long-term potential difference. At the same time, the ‘Alzheimer's disease’ pathway is also enriched in PD (Fig. 4), demonstrating some commonality in AD and PD, which suggests neurons face same challenges in NDs.

By screening for Fr-DEGs in different cells, we attempted to construct a ferroptosis-related map of all cell types in PD. Among the identified genes, we found a decrease in GPX4 among the ferroptosis-associated DEGs in dopaminergic neurons. GPX4 encodes phospholipid hydroperoxide glutathione peroxidase (PHGPx), which possesses membrane lipid hydroperoxide scavenging ability^27^. Inactivation of GPX4 in cells triggers the accumulation of lipid peroxides, leading to ferroptosis^28^. FTH is a ubiquitous cellular iron storage protein. When cellular iron levels are stable, a decrease in FTH will lead to an increase in the catalytic metal, ultimately resulting in the generation of ROS via Fenton chemistry^29^. Such increases in lipid peroxidation and free iron support our hypothesis that dopaminergic neurons are at a greater risk of ferroptosis and may be lost as a result. Endothelial cells and pericytes demonstrated the most ferroptosis-related DEGs, which may be related to their key function in forming the blood–brain barrier and maintaining brain iron homeostasis. In the pathological progression of PD, the impairment of the blood–brain barrier function may lead to persistent disorders in brain iron homeostasis, thereby further promoting the development of PD.

We conjectured that, if ferroptosis is closely related to the loss of dopaminergic neurons, it would follow that the secretion of ferroptosis-related proteins between cells affects the sensitivity of dopaminergic neurons to ferroptosis. To investigate this possibility, we explored whether proteins expressed by ferroptosis-associated DEGs were secreted, identifying three such genes: HMGB1, CP and TF. The high-mobility group box 1 protein encoded by HMGB1 is a highly abundant and conserved protein with important intracellular and extracellular biological activities^30^. In the nucleus, HMGB1 can interact with DNA and histones to maintain chromosome structure and function, while, in the cytoplasm, HMGB1 can combine with Beclin 1 protein to promote autophagy. Extracellularly, HMGB1 can regulate inflammation and immune response. The secretion and release of HMGB1 is not only regulated by post-translational modification, but also promoted by ferroptosis^31^. Knockdown of HMGB1 has been shown to reduce erastin-induced ferroptosis^32^, although neuronal cells have not been examined in this context. Anti-HMGB1 antibodies also elicited neuroprotective effects in a PD rat model^33^. In the SN of PD patients, HMGB1 showed an overall trend of increased expression, which may increase the sensitivity of PD patient dopaminergic neurons to ferroptosis.

Ceruloplasmin (CP) and transferrin (TF) are molecules that directly participate in iron metabolism and are critical for brain iron homeostasis. CP is a ferroxidase enzyme mainly expressed in a unique subpopulation of astrocytes that surround capillaries^34,35^. A clinical study showed that it is discrepancy for serum ceruloplasmin (CP) levels in PD patients, and that serum CP reduction is more easily to accelerate the progression of PD than that of serum CP normal patients ^36^, which might be the same as the global *Cp* gene deficiency related parkinsonism symptoms in aceruloplasminemia patients and *Cp*^*-/-*^ mice ^37,38^. However, CP in the brain plays a different critical role, and the tissue-specific expression may reveal the complexity and precision of life. The concentration of CP in serum is 300–450 μg/mL and CP in cerebrospinal fluid is only 1 μg/ mL^39^, and increased CP in different brain regions has been observed in NDs^40^, therefore, the role of CP in brain and its relationship with PD might be different. We verified the elevated expression of CP content in the bulk RNA-seq set. A study examining the variation and localization of the CP gene in PD showed that CP colocalizes with Lewy bodies in PD^41^. Our study has recently shown that astrocytic *Cp* knockout can improve learning and memory in aged mice due to the decreased iron contents in the brain^42^. These data confirmed the critical role of elevated brain CP protein in the development of PD, which is different from CP in peripheral serum. TF is an iron transport protein that delivers the metal to most cells for endocytic uptake of the metal^43^. Decreased TF protein levels have been reported in the SN of post-mortem PD brains^44^. The decreased expression of TF in oligodendrocytes in the SN of PD patients may decrease the ability of oligodendrocytes to sequester excess iron, ultimately resulting in an increase in oxidative stress in dopaminergic neurons.

It is important to note some limitations of our study. The bulk RNA-seq data from the GEO database verified the increase in CP mRNA expression. It has been reported that the expression of CP protein was not changed obviously in SN or cortex and that CP activity decreased in SN of PD brain^45^. We speculate that the reason for this phenomenon may be that CP in the microvascular lumen affects the determination of CP in the brain. Therefore, the possible mechanism of CP’s effect on PD has not been fully explored in the brain. For the specific role and pathogenic mechanisms relating CP, HMGB1 and TF to PD, further exploration and verification are still required. Interestingly, men are twice as likely to develop PD as women, however women have a higher mortality rate from PD with a more rapid disease progression^46^. Future studies should also take into account the potential influence of gender on iron metabolism and its participation in PD pathogenesis and progression.

## Conclusion

In summary, we used the single-cell RNA-seq data from PD SN to analyze ferroptosis-related differentially expressed genes, revealing a correlation between the different types of cells in the SN of PD patients and ferroptosis. Notably, three differentially expressed genes (HMGB1, CP, TF), at the single-cell level, encode secreted proteins which may impact the susceptibility of dopaminergic neurons in the SN of PD patients to ferroptosis. In addition, we verified the elevated expression of CP in the PD brain through a bulk RNA-seq set from both the GEO data set, identifying CP as a potential therapeutic target for neuroprotection in PD.

## Data Availability

The datasets [GENERATED] for this study can be found in the [GEO DATABASE] [https://www.ncbi.nlm.nih.gov/geo/].
